# BLV-CoCoMo-qPCR: a useful tool for evaluating bovine leukemia virus infection status

**DOI:** 10.1186/1746-6148-8-167

**Published:** 2012-09-21

**Authors:** Mayuko Jimba, Shin-nosuke Takeshima, Hironobu Murakami, Junko Kohara, Naohiko Kobayashi, Tamako Matsuhashi, Takashi Ohmori, Tetsuo Nunoya, Yoko Aida

**Affiliations:** 1 Viral Infectious Diseases Unit, RIKEN, 2–1 Hirosawa, Wako, Saitama 351-0198, Japan; 2 Laboratory of Viral Infectious Diseases, Department of Medical Genome Sciences, Graduate School of Frontier Science, The University of Tokyo, Wako, Saitama 351-0198, Japan; 3 Japan Foundation for AIDS Prevention, Chiyoda-ku, Tokyo, 101-0061, Japan; 4 Animal Research Center, Hokkaido Research Organization, Shintoku, Hokkaido, 080-0038, Japan; 5 Gifu Prefectural Livestock Research Institute, 4393–1 Makigahora, Kiyomi, Takayama, Gifu, 506-0101, Japan; 6 Nippon Institute for Biological Science, 9-2221-1 Shinmachi Ome, Tokyo, 198-0024, Japan

**Keywords:** Bovine leukemia virus, Real-time PCR, Proviral load, Serological test, Experimental infection

## Abstract

**Background:**

Bovine leukemia virus (BLV) is associated with enzootic bovine leukosis, which is the most common neoplastic disease of cattle. BLV infects cattle worldwide, imposing a severe economic impact on the dairy cattle industry. Recently, we developed a new quantitative real-time polymerase chain reaction (PCR) method using Coordination of Common Motifs (CoCoMo) primers to measure the proviral load of known and novel BLV variants in BLV-infected animals. Indeed, the assay was highly effective in detecting BLV in cattle from a range of international locations. This assay enabled us to demonstrate that proviral load correlates not only with BLV infection capacity as assessed by syncytium formation, but also with BLV disease progression. In this study, we compared the sensitivity of our BLV-CoCoMo-qPCR method for detecting BLV proviruses with the sensitivities of two real-time PCR systems, and also determined the differences of proviral load with serotests.

**Results:**

BLV-CoCoMo-qPCR was found to be highly sensitive when compared with the real-time PCR-based TaqMan MGB assay developed by Lew *et al*. and the commercial TaKaRa cycleave PCR system. The BLV copy number determined by BLV-CoCoMo-qPCR was only partially correlated with the positive rate for anti-BLV antibody as determined by the enzyme-linked immunosorbent assay, passive hemagglutination reaction, or agar gel immunodiffusion. This result indicates that, although serotests are widely used for the diagnosis of BLV infection, it is difficult to detect BLV infection with confidence by using serological tests alone. Two cattle were experimentally infected with BLV. The kinetics of the provirus did not precisely correlate with the change in anti-BLV antibody production. Moreover, both reactions were different in cattle that carried different bovine leukocyte antigen (BoLA)-DRB3 genotypes.

**Conclusions:**

Our results suggest that the quantitative measurement of proviral load by BLV-CoCoMo-qPCR is useful tool for evaluating the progression of BLV-induced disease. BLV-CoCoMo-qPCR allows us to monitor the spread of BLV infection in different viewpoint compared with classical serotest.

## Background

Bovine leukemia virus (BLV) is associated with enzootic bovine leucosis (EBL) [1], which is the most common neoplastic disease of cattle. Infection by BLV can remain clinically silent, with cattle in an aleukemic state. Alternatively, it can emerge as persistent lymphocytosis (PL), characterized by an increased number of B lymphocytes, and, more rarely, as B-cell lymphomas in various lymph nodes after a long latent period [2]. Sheep that are experimentally inoculated with BLV develop B-cell tumors at a higher frequency and with a shorter latent period than naturally infected cattle [2,3].

BLV is closely related to human T-cell leukemia virus types 1 and 2 (HTLV-1 and −2), which are associated with adult T-cell leukemia (ATL) and with the chronic neurological disorder tropical spastic paraparesis/HTLV-1-associated myelopathy [2]. Defective HTLV-1 proviral genomes have been found in more than half of all examined patients with ATL [4-7]. By contrast, genomic Southern hybridization of BLV proviral DNA yielded only bands that corresponded to the full-size provirus in all 23 cattle at the lymphoma stage and in all 7 BLV-infected but healthy cattle [8]. Polymerase chain reaction (PCR) with primers located in the long terminal repeat (LTRs) clearly demonstrated that almost the complete provirus was retained in all 27 cattle with lymphomas and in all 19 infected but healthy cattle [8]. We previously performed conventional PCR with various primers spanning the entire BLV genome to detect even small defects. The obtained PCR products specifically covered the entire BLV genome in all 40 of the BLV-infected cattle tested [8]. Therefore, it appears that at least one copy of the full-length BLV proviral genome was maintained in each animal throughout the course of the disease. Moreover, either large or small deletions of proviral genomes may be very rare events in BLV-infected cattle.

The above findings suggest that the BLV provirus remains integrated in cellular genomes [9,10], even in the absence of detectable BLV antibodies. After their infection, BLV expression in cattle is blocked at the transcriptional level during the latent period [11]. This repression appears to be very important in the escape of BLV from the host immunosurveillance system. Such silencing is also observed in BLV-infected sheep [11,12]. The mechanism responsible for BLV silencing is unknown. Therefore, in addition to the routine diagnosis of BLV infection using conventional serological techniques such as the agar gel immunodiffusion (AGID) [3,13-15] and enzyme-linked immunosorbent assay (ELISA) [14-17], diagnostic BLV PCR techniques that aim to detect the integrated BLV proviral genome within the host genome are also commonly used [8,14-16,18,19].

TaKaRa cycleave PCR was recently developed as a commercial BLV detection kit targeting the *tax* region, which is present at only one copy per provirus, and encodes a transactivator protein Tax. Lew *et al.*[20] reported a method to quantify BLV provirus using real-time PCR. Their method targets the BLV *pol* gene, which is present at only one copy per provirus, and the primer annealing regions are potentially susceptible to mutation. We recently developed a new quantitative real-time PCR (qPCR) method targeting the BLV LTR. This region is present at two copies per provirus, which contributes to the improved sensitivity of our assay [21]. To design degenerate primers addressing BLV diversity, our BLV-CoCoMo-qPCR method uses the Coordination of Common Motifs (CoCoMo) algorithm, which was developed especially for the detection of multiple viral species. The obtained primers were used to measure the proviral loads of known and novel BLV variants in clinical animals. This method was highly effective in detecting a wide range of mutated BLV viruses in cattle from various international locations. BLV infects cattle worldwide, imposing a severe economic impact on the dairy cattle industry [13-16,22,23]. To normalize the viral genomic DNA, the BLV-CoCoMo-qPCR technique amplifies a single-copy host gene, the *bovine leukocyte antigen (BoLA)-DRA* gene, in parallel with the viral genomic DNA. This measurement permits adjustment for variations in amplification efficiency between samples. Thus, the assay is specific, sensitive, quantitative, and reproducible, and is able to detect BLV strains from cattle worldwide, including those for which previous attempts at detection by nested PCR have failed. Using this assay, we previously demonstrated that proviral load correlates not only with BLV infection capacity as assessed by syncytium formation, but also with BLV disease progression.

In this study, we compared the sensitivity of our BLV-CoCoMo-qPCR method for detecting BLV proviruses with the sensitivities and reproducibilities of two real-time PCR systems, using an infectious full-length molecular clone of BLV, pBLV-IF [24]. The sensitivities of antibody-detection methods such as ELISA, passive hemagglutination reaction (PHA), and AGID, and the proviral load estimated by BLV-CoCoMo-qPCR were estimated in 370 cattle. To analyze the kinetics of the provirus and relevance of the BLV antibody, two BLV-negative Holstein-Friesian cattle that carried different *BoLA-DRB3* genotypes were experimentally infected with BLV, and the titers of serum antibody and proviral load were measured.

## Methods

### Animal samples and isolation of genomic DNA and serum

Blood samples were obtained from 48 Japanese black cattle in herd A and 322 Holstein-Friesian cattle in herd B. These cattle were all maintained in Japan. For experimental infection, two BLV-negative one-year-old Holstein-Friesian cattle were used. Genomic DNAs for PCR amplification were isolated from EDTA-treated whole blood samples by using the Wizard Genomic DNA Purification Kit (Promega Corporation, Tokyo, Japan). The Sera were separated from blood of cattle mentioned above.

### Detection of BLV provirus by real-time PCR

Real-time PCR was performed with TaqMan Universal Master Mix II (Life Technologies, Tokyo, Japan) for BLV-CoCoMo-qPCR [21] and the TaqMan minor groove binder (MGB) assay developed by Lew *et al.*[20] or with the Cycleave PCR system (TaKaRa Bio, Inc, Tokyo, Japan) on the 7500 FAST Real-time PCR System (Life Technologies).

The BLV LTR genes were detected by BLV-CoCoMo-qPCR [21]. In brief, 120-bp of the BLV-LTR gene were amplified by the CoCoMo6 and CoCoMo81 primer set and detected with 15 bp of the 6-carboxyfluorescein (FAM)-labeled MGB probe. The BLV *pol* gene was detected by the TaqMan MGB assay developed by Lew *et al.*[20]. Briefly, 67 bp of the BLV *pol* gene were amplified by the BLVMGBF and BLVMGBR primer set and detected with 15 bp of the FAM-labeled MGB probe. The BLV *tax* gene was detected as suggested by the manufacturer, using the Cycleave PCR BLV detection kit (TaKaRa Bio Inc.), which amplified the BLV *tax* gene and detected it with the FAM-labeled Cycleave probe.

### Evaluation of BLV proviral load by BLV-CoCoMo-qPCR

The proviral load (expressed as the number of copies of provirus per 100,000 peripheral blood mononuclear cells [PBMCs]) was evaluated by qPCR on the genomic DNA for the numbers of copies of LTR and *BoLA-DRA*[21].

In brief, 30 ng of cattle genomic DNA, which usually contained 1 x 10^3^ to 3 x 10^3^ copies of *BoLA-DRA* genes (0.5 to 1.5 x 10^3^ of cell number), was used for PCR amplification. BLV copy number were calculated using 10 to 1 x 10^6^ copies of the standard plasmid, which contained the BLV-LTR region inserted into pBluescript II SK + plasmid. Each value was calculated in a single experiment.

### Detection of BLV provirus by nested PCR

BLV LTR gene was detected by nested PCR, as described previously [21]. In brief, the first PCR amplification was performed with the primers BLTRF-YR and BLTRR. The first PCR amplicons were subsequently applied to the second PCR, with the 256 and 453 primer set. PCR amplification was performed with a TGRADIENT thermocycler (Biometra). PCR products were detected by ethidium bromide staining.

### Detection of anti-BLV antibody in serum samples

Anti-BLV antibodies were detected using three detection systems. The PHA method was performed according to the manufacturer’s instructions using the Bovine Leucosis Antibody Assay Kit “Nisseiken” (Nisseiken, Tokyo, Japan). The AGID test for detection of the anti-BLV antibody was performed according to the manufacturer’s instructions, using a commercial bovine leukemia virus antibody test kit (Kitazato, Japan). Finally, ELISA was performed according to the manufacturer’s instructions, using an ELISA kit for detecting anti-BLV antibody (JNC Inc., Tokyo, Japan).

### Experimental infection of BLV

Two BLV-negative one-year-old Holstein-Friesian cattle (SK576 and SK577) were experimentally challenged subcutaneously with 0.5 ml of blood obtained from BLV-seropositive Japanese Black cattle (16-year-old, normal lymphocyte count [4,660/μl]). Blood samples (used for DNA and serum isolation) were collected weekly for 10 weeks after the first inoculation. BLV proviral loads were measured by BLV-CoCoMo-qPCR. The S/P values of ELISA were determined by: S/P = [(absorbance of antigen existence and sample added well)-(absorbance of antigen absence and sample added well)]/[(absorbance of antigen existence and positive control added well)-(absorbance of antigen absence and positive control added well)]. The dilution ratio of PHA indicates the observed limit point of hemagglutination.

### BoLA-DRB3 typing

*BoLA-DRB3* alleles were typed by the PCR-sequence based typing (SBT) method [25]. In brief, *BoLA-DRB3* exon 2 was amplified by the DRB3FRW and DRB3REV primer set by single-step PCR, and the nucleotide sequences were subsequently determined. Sequence data were analyzed by ASSIGN 400 ATF software (Conexio Genomics, Fremantle, Australia), and both *BoLA-DRB3* alleles of the cattle were determined.

## Results

A comparison of the sensitivity and the reproducibility of BLV-CoCoMo-qPCR to those of other TaqMan real-time PCR methods for the detection of the BLV provirus.

We compared the sensitivity and the reproducibility of the BLV-CoCoMo-qPCR method with those of two real-time PCR systems for BLV provirus detection: the TaqMan MGB assay developed by Lew *et al.*[20], and the commercial TaKaRa cycleave PCR assay. This experiment was performed with an infectious full-length molecular clone of BLV, pBLV-IF [24] (Table 1). 

**Table 1:** Comparison of BLV proviral detection by BLV-CoCoMo-qPCR, the TaqMan MGB assay, and TaKaRa cycleave PCR

**pBLV-IF**	**CoCoMo-qPCR**^**a**^	**Lew Method**^**b**^	**TaKaRa**
**(copy number)**			**Cycleave PCR**^**c**^
100	3/3^d^	3/3	3/3
50	3/3	3/3	3/3
25	3/3	3/3	3/3
12.5	3/3	3/3	3/3
6.25	3/3	3/3	3/3
3.125	3/3	3/3	3/3
1.5625	3/3	0/3	3/3
0.78125	3/3	0/3	2/3
0	0/3	0/3	0/3

^a^ BLV LTR genes were detected by BLV-CoCoMo-qPCR (Jimba *et al.*, 2010).

^b^ BLV *pol* gene was detected by TaqMan MGB assay developed by Lew *et al.* (Lew *et al.*, 2004).

^c^ BLV *tax* gene was detected by the cycleave PCR BLV detection kit (TaKaRa Bio inc.).

^d^ Number detected per number tested.

To determine the sensitivity, we performed a 2-fold dilution of pBLV-IF^conc^ and multifold dilutions of pBLV-LTR^conc^ to give a range of provirus copy numbers from 100 to 0.78125, and examined after triplicate PCR amplifications the percentage of successful amplification. All of the amplifications obtained by the three methods successfully detected BLV-IF when it was present at ≥3.125 copies. The sensitivities of the three real-time PCR methods for the detection of pBLV-IF differed when ≤1.5625 copies of the provirus was employed. The BLV-CoCoMo-qPCR and TaKaRa cycleave PCR methods, but not the TaqMan MGB assay developed by Lew *et al.* successfully detected pBLV-IF with 1.5625 copies of provirus at a rate of 100%. With 0.78125 copies of the pBLV-IF provirus, the detection rate was significantly lower with the TaqMan MGB assay developed by Lew *et al.* (0%), but BLV-CoCoMo-qPCR (100%) and TaKaRa cycleave PCR (66.7%) resulted in high and moderate detection rates. Together, these results indicate that, at low proviral loads of pBLV-IF, the sensitivity of BLV-CoCoMo-qPCR is better than those of the TaqMan MGB assay developed by Lew *et al.* and the TaKaRa cycleave PCR assay.

**Figure 1:**
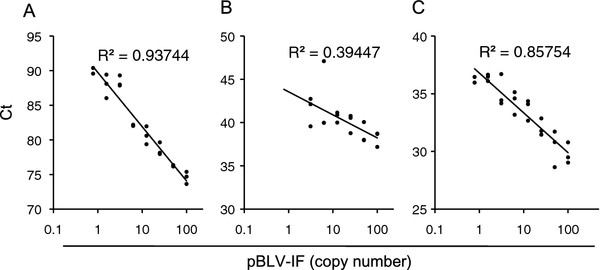
** Sensitivity and reproducibility of each real-time PCR method using a pBLV-IF. **The copy number of pBLV-IF was determined by calculation and TaKaRa Cycleave PCR. One hundred copy of pBLV-IF was diluted 2-fold to construct the standard curve. Threshold values (Ct) were plotted against corresponding pBLV-IF copy numbers and the correlation coefficient (R^2^) was determined. The experiments were run in duplicate and independently repeated three times with the same dilutions. pBLV-IF standard curves were generated by using the results of CoCoMo-qPCR (**A**), the TaqMan MGB assay developed by Lew *et al.* (**B**), and TaKaRa Cycleave PCR (**C**).

Next, we evaluated the reproducibility of the copy numbers obtained by the three methods (Figure 1). At low copies numbers, the copy number determined by CoCoMo-qPCR was the most reproducible (R^2^ = 0.93744), the copy number determined by TaKaRa cycleve PCR was moderately reproducible (R^2^ = 0.85754), and the copy number detected by the TaqMan MGB assay developed by Lew *et al.* was the least reproducible (R^2^ = 0.39447). These results clearly demonstrated that this assay has good reproducibility.

Thus, it appeared that BLV-CoCoMo-qPCR was the most suitable method for estimating BLV proviral load.

### Comparison of BLV-CoCoMo-qPCR with nested PCR and serological tests

**Figure 2:**
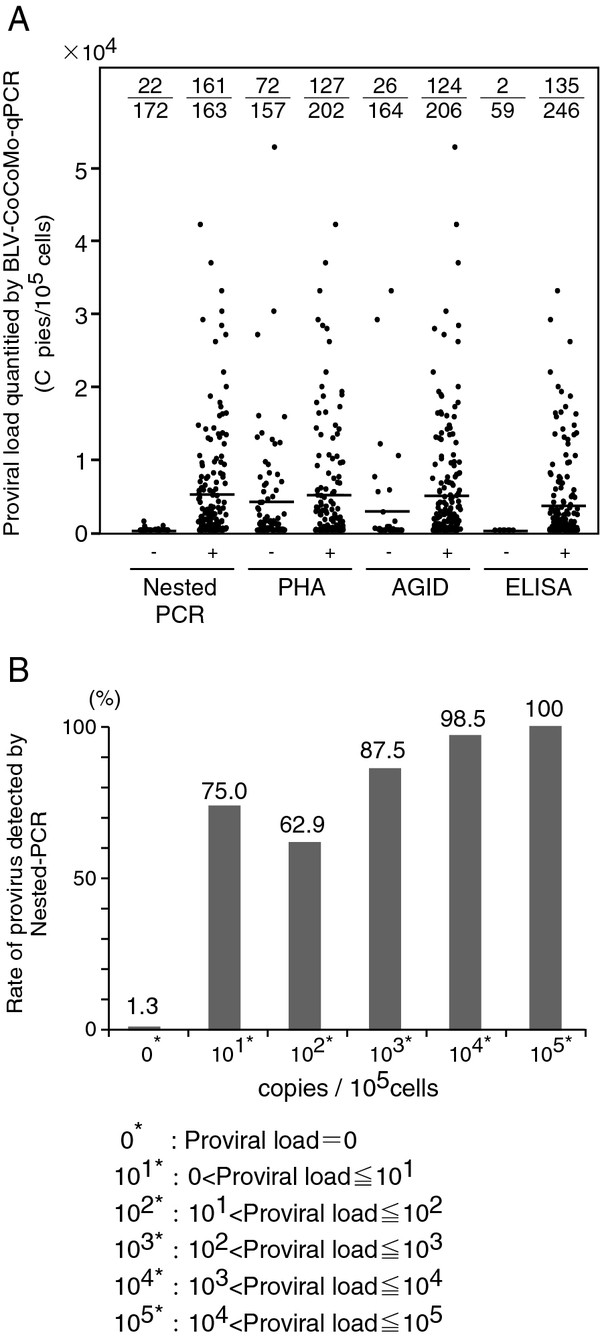
** Comparison of BLV detection by BLV-CoCoMo-qPCR, nested PCR, and serological tests. **(**A**) BLV proviral load, as evaluated by BLV-CoCoMo-qPCR, in whole blood from 370 cattle that were either positive (+) or negative (−) for BLV LTR sequences, as determined by nested PCR, and serological tests such as the passive hemagglutination reaction (PHA), agar gel Immunodiffusion (AGID) and enzyme-linked immunosorbent assay (ELISA). Bars show the median BLV proviral load values. The actual number of cattle that were positive by BLV-CoCoMo-qPCR alone per number of cattle that were either positive (+) or negative (−) for BLV infection as determined by each test is indicated at the upper of each block. (**B**) Nested PCR positive rates for different proviral copy numbers per 10^5^ cells (copy number of 0-10^5^), as evaluated by BLV-CoCoMo-qPCR. The positive rate was 1.3 % when the proviral load was 0.

We compared the sensitivity of BLV-CoCoMo-qPCR with those of nested PCR and conventional serological techniques, including AGID, PHA, and ELISA, using 370 clinical samples from two farms in Japan (Table 2 and Figure 2).

**Table 2:** Comparison of BLV detection methods using 370 cattle

**(a) Copy number/10**^**5**^** cells by BLV-CoCoMo-qPCR=0**					
**Animal no.**	**Copy number**^**a**^	**Nested PCR**	**PHA**^**b**^	**AGID**^**c**^	**ELISA**^**d**^
1-39	0	-^e^	-	-	-
40-53	0	-	+^f^	-	-
54-85	0	-	-	-	+
86	0	-	-	+	-
87-123	0	-	+	-	+
124-129	0	-	-	+	+
130-138	0	-	+	+	+
139-141	0	-	-	+	NT^g^
142	0	-	NT	-	-
143	0	-	NT	-	+
144	0	-	NT	-	NT
145-148	0	-	+	-	NT
149,150	0	-	+	+	NT
151,152	0	NT	-	-	+
153	0	NT	-	+	+
154	0	NT	+	-	-
155	0	NT	+	+	NT
156	0	NT	+	-	NT
157-159	0	NT	+	-	+
160,161	0	NT	+	+	+
162	0	+	-	-	+
163	0	+	+	-	-
-	163	150	85	138	57
+	0	2	75	25	94
Tested	163	152	160	163	151
**(b) Copy number/105 cells by BLV-CoCoMo-qPCR>0**					
164	1	**-**	+	+	+
165	1	+	+	-	+
166	1	-	-	-	-
167	2	+	-	+	+
168	3	+	+	+	+
169	3	NT	+	-	+
170	4	+	-	+	+
171	5	-	+	-	-
172	6	+	+	+	NT
173	8	+	+	-	+
174	9	-	+	-	+
175	9	+	-	-	+
176	9	NT	+	+	NT
177	10	+	-	+	+
178	12	+	+	+	+
179	15	+	-	+	NT
180,181	16,17	+	-	+	+
182	17	+	+	+	+
183	17	NT	+	+	+
184	18	-	+	+	+
185	19	+	-	+	+
186,187	20,21	-	+	+	+
188	22	+	-	+	+
189	23	-	-	-	+
190	25	+	+	+	+
191	27	-	-	+	+
192	33	+	-	+	+
193	37	-	-	+	+
194	39	-	+	+	+
195	40	-	+	+	NT
196	41	+	-	+	+
197	42	+	+	+	+
198,199	43,44	+	-	+	+
200	45	-	+	-	+
201	49	+	+	+	+
202	50	-	+	+	+
203	53	+	+	+	+
204	55	NT	+	+	+
205	58	-	+	+	+
206	67	+	+	+	+
207	68	NT	-	+	+
208–210	71,74,79	+	+	+	+
211	83	-	NT	+	+
212	85	+	-	+	+
213	91	NT	-	+	+
214,215	92,96	+	+	+	NT
216	99	-	-	+	+
217	110	+	+	-	+
218	110	-	-	+	+
219	110	+	+	+	NT
220	145	+	+	-	+
221	145	+	+	+	+
222	149	-	-	+	+
223	154	+	NT	+	NT
224	166	+	+	+	+
225	166	+	-	+	NT
226	176	NT	-	+	+
227	176	+	+	-	+
228	180	-	+	+	NT
229,230	190,194	+	+	+	NT
231	202	NT	+	+	+
232	203	+	+	+	+
233	231	+	-	-	+
234	254	+	+	+	+
235	261	+	+	-	+
236	265	+	NT	+	+
237	270	+	+	+	+
238	295	+	-	+	+
239	321	+	+	+	NT
240	347	+	+	+	+
241	349	NT	+	+	+
242,243	363,366	+	+	+	+
244	374	+	-	+	+
245	472	+	+	-	+
246	475	+	+	+	NT
247	491	+	+	-	+
248	565	-	-	+	+
249	605	+	+	+	NT
250	634	-	+	+	+
251,252	664,684	+	-	+	+
253,254	705,710	+	+	+	+
255-257	835,868,875	+	-	+	+
258	876	+	+	+	+
259	942	+	-	+	NT
260,261	1088,1170	+	-	+	+
262	1196	+	+	+	NT
263	1219	+	-	+	+
264,265	1223,1225	+	+	+	+
266	1233	-	-	-	NT
267,268	1279,1282	+	-	+	+
269	1445	+	NT	+	+
270	1459	+	+	+	NT
271	1495	+	-	+	+
272,273	1501,1567	+	+	+	+
274	1648	+	-	+	+
275	1671	+	+	+	+
276	1793	+	+	+	NT
277	1844	+	-	+	NT
278	1902	+	+	+	NT
279	2068	+	-	+	+
280-282	2130,2142, 2183	+	+	+	+
283	2218	-	NT	+	+
284	2219	+	-	+	+
285	2326	+	NT	+	+
286	2338	+	+	+	+
287	2524	+	+	-	+
288	2616	+	+	+	NT
289,290	2621,2727	+	+	+	+
291	2849	NT	+	+	+
292	2855	+	-	+	+
293	2963	+	+	+	+
294	3062	+	-	+	+
295,296	3097,3192	+	+	+	+
297	3294	+	+	+	NT
298	3512	NT	+	+	+
299	3619	NT	+	+	NT
300	3833	+	-	+	+
301	3859	NT	-	+	NT
302	3871	+	+	+	+
303	3955	+	+	+	+
304	4291	+	-	+	NT
305	4517	+	+	+	+
306	4623	NT	-	+	+
307,308	4774,4837	+	+	+	+
309,310	5079,5291	+	+	+	NT
311	5296	NT	-	-	NT
312	5453	+	+	+	NT
313	5490	+	-	+	NT
314,315	5551,5667	+	+	-	+
316	5821	+	+	+	+
317,318	6188,6423	+	-	+	NT
319	6471	NT	+	+	+
320	6567	+	+	+	+
321	6686	+	-	+	+
322	7127	+	+	+	+
323	7303	+	-	+	NT
324	7371	+	+	-	+
325	7376	+	NT	+	+
326	7665	+	-	+	+
327	7835	+	+	+	NT
328	7903	+	-	+	+
329	8879	+	+	+	NT
330	9048	+	-	+	+
331,332	9275,9358	+	+	+	+
333	9417	+	-	+	NT
334,335	9843,10154	+	+	+	NT
336	10252	+	+	-	+
337	10363	NT	+	+	+
338	11874	+	-	-	+
339	12040	NT	-	+	NT
340	12461	+	-	+	+
341	12667	+	+	+	NT
342	12791	+	-	+	+
343,344	13136,13229	+	+	+	+
345	13379	+	-	+	+
346	13904	+	+	+	+
347	14057	+	+	+	NT
348	14433	+	+	+	+
349	15602	NT	-	+	+
350	15747	+	-	+	NT
351	15982	+	NT	+	+
352	16099	+	+	+	+
353	16220	NT	+	+	+
354	16984	+	+	+	+
355	17577	+	+	+	NT
356	18419	+	+	+	+
357	18624	NT	+	+	NT
358	19043	NT	+	+	+
359-361	19732,21744 25912	+	+	+	+
362	26883	+	-	+	NT
363	27719	NT	+	+	NT
364	28154	+	+	+	NT
365	28934	+	+	-	+
366	30098	+	-	+	NT
367	32909	+	+	-	+
368,369	36753,42015	+	+	+	NT
370	52680	NT	-	+	NT
-	0	22	72	26	2
+	208	161	127	181	152
Tested	208	183	199	207	154

^a^Copy number/10^5^cells by BLV-CoCoMo-qPCR.

^b^PHA, Passive hemagglutination antigen method performed with the Bovine Leucosis antibody assay kit “Nisseiken”.

^c^AGID, Agarose gel Immuno-diffusiontest was also performed for anti-BLV antibody detection with a commercial bovine leukemia virus antibody test kit.

^d^ELISA, Enzyme-linked immunosorbent assay was performed with an ELISA kit for detecting anti-BLV antibody.

^e^-, Negative.

^f^+, Positive.

^g^NT, Not test.

A total of 39 out of 370 cattle were negative for BLV provirus and anti-BLV antibody, as determined by the four methods. A total of 150 out of 370 cattle were negative for BLV provirus, as determined by both BLV-CoCoMo-qPCR and nested PCR. However, some animals that were negative for proviral load, as determined by BLV-CoCoMo-qPCR, were positive in the serological tests. For example, 75 of 160 samples (46.9%) were positive by PHA, 25 of 163 samples (15.3%) were positive by AGID, and 94 of 151 samples (62.3%) were positive by ELISA.

Furthermore, a total of 56 out of 370 cattle were positive for BLV provirus and anti-BLV antibody, as determined by all five methods. A total of 161 out of 370 cattle were positive for BLV provirus, as determined by both BLV-CoCoMo-qPCR and nested PCR. By contrast, a total of 22 out of 183 cattle (12.0%) were positive for BLV provirus by BLV-CoCoMo-qPCR but were negative in the nested PCR assay. However, the positive rate for nested PCR was 100% in animals with proviral loads of >1,500 copies per 10^5^ cells, indicating that the positive rate for nested PCR in animals correlated well with the level of proviral load determined by BLV-CoCoMo-qPCR. Moreover, some animals that were positive for proviral load, as determined by BLV-CoCoMo-qPCR were positive in the serological tests: 127 of 199 samples (63.8%) were positive by PHA, 181 of 207 samples (87.4%) were positive by AGID, and 152 of 154 samples (98.7%) were positive by ELISA.

Figure 2A shows the proviral loads of samples that were either negative or positive by nested PCR, PHA, AGID, and ELISA. A total of 163 cattle were positive for BLV LTR sequences as determined by nested PCR, with copy numbers ranging from 0 to 42,015 copies per 10^5^ cells (mean 5,135 copy). By contrast, 22 cattle were negative by nested PCR but were positive by BLV-CoCoMo-qPCR, with proviral loads ranging from 0 to 1,233 copies per 10^5^ cells (mean 20 copy). A total of 202 samples were positive for anti-BLV antibody as determined by PHA, with copy numbers ranging from 0 to 42,015 copies per 10^5^ cells (mean 3,427 copies). By contrast, 157 cattle were negative for anti-BLV antibody as determined by PHA but were positive as determined by BLV-CoCoMo-qPCR, with proviral loads ranging from 0 to 52,680 copies per 10^5^ cells (mean 2,049 copies). A total of 206 samples were positive for anti-BLV antibody by AGID, with copy numbers ranging from 0 to 52,680 copies per 10^5^ cells (mean 4,516 copies). By contrast, 164 cattle were negative for anti-BLV antibody by AGID but positive by BLV-CoCoMo-qPCR, with proviral loads ranging from 0 to 32,909 copies per 10^5^cells (mean 693 copies). A total of 246 samples were positive for anti-BLV antibody by ELISA, with copy numbers ranging from 0 to 32,909 copies per 10^5^ cells (mean 2,380 copies). By contrast, 59 cattle were negative for anti-BLV antibody by ELISA but positive by BLV-CoCoMo-qPCR, with proviral loads ranging from 0 to 5 copies per 10^5^ cells (mean 0.1 copies). Moreover, Figure 2A indicated that the proportion of animals that was negative for anti-BLV antibodies by serological tests but positive by BLV-CoCoMo-qPCR was higher than the proportion that was negative for provirus detection by nested PCR but positive by BLV-CoCoMo-qPCR. These results clearly demonstrate that the number of animals that were positive for the BLV antibody by the three serological tests did not correlate with the proviral loads determined by BLV-CoCoMo-qPCR.

We next calculated the positive rate for the nested PCR method in animals with BLV proviral copy numbers of 0 to 10^5^ per 10^5^ cells, as evaluated by BLV-CoCoMo-qPCR (Figure 2B). The proviral copy numbers of 152 samples were estimated to be “0” by BLV-CoCoMo-qPCR. Two of the 152 samples (1.3%) were positive, but 150 samples (98.7%) were negative for BLV LTR by nested PCR. Positive rates for the nested PCR ranged from 62.9% to 98.5% among animals with proviral copy numbers ranging from 10^0^ to 10^4^ copies per 10^5^ cells. The positive rate for nested PCR was 100% in animals with high proviral loads (>10^4^ copies per 10^5^ cells). Thus, the positive rate for the nested PCR in animals correlated well with the level of proviral load determined by BLV-CoCoMo-qPCR.

### Kinetics of proviral load and detection of antibodies in cattle experimentally infected with BLV

**Figure 3:**
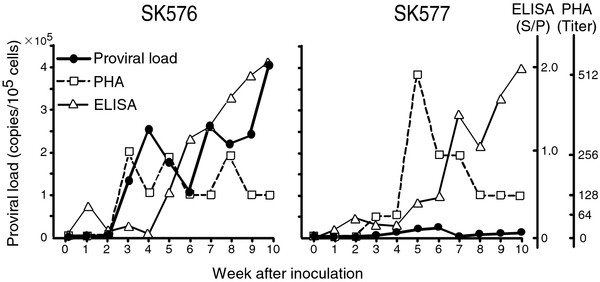
** Proviral load and antibody titer in two experimentally challenged cattle. **Two BLV-negative one-year-old Holstein-Friesian cattle (K576 and K577) were experimentally challenged subcutaneously with 0.5 ml of blood obtained from BLV-seropositive Japanese Black cattle. Blood and serum samples were obtained weekly for 10 weeks after the first inoculation with infected blood. BLV proviral loads were measured by BLV-CoCoMo-qPCR. Anti-BLV antibodies were detected by PHA and ELISA. Graphical representations of proviral load, ELISA S/P values, and PHA titer are shown.

Our results showed an inconsistency between the proviral load as evaluated by BLV-CoCoMo-qPCR and the detection of BLV infection by serological tests. To investigate the reasons for these different results, two cattle were experimentally infected with BLV, and the anti-BLV antibody titer in the serum and proviral load were examined (Figure 3). Polymorphisms in *BoLA* class II genes are responsible for the outcomes of infectious diseases such as neosporosis, Lone Star tick, clinical mastitis, and enzootic bovine leukosis [26-30]. Therefore, the two cattle (SK576 and SK577) were genotyped for *BoLA-DRB3* alleles by the PCR-SBT method. SK576 carried alleles DRB3*0101/1201, and SK577 carried alleles DRB3*14011/1201. 

We evaluated the number of BLV-infected cells in the two cattle by BLV-CoCoMo-qPCR from 1 to 10 weeks after experimental infection with BLV, and compared the production of anti-BLV antibodies by PHA and ELISA. In Figure 3, the titers indicate the reverse of the serum dilution for which 50% inhibition of PHA was observed. The ELISA S/P values were measured for 10 weeks. In SK577, although the BLV copy number was maintained at low levels for 10 weeks after BLV challenge, the ELISA S/P values increased gradually throughout the experimental period after 5 weeks. The PHA titer reached a peak at 5 weeks after BLV challenge and then decreased. In SK576, the BLV copy number per 10^5^ cells increased gradually throughout the experimental period. The ELISA S/P value started to increase gradually at 5 weeks, and the value remained high for the experimental period. Interestingly, the PHA titer was lower at the high viral load stage and was higher at the low viral load stage.

## Discussion

Recently, we developed the BLV-CoCoMo-qPCR system to detect various BLV strains with broad geographical origins. Proviral load determined in this manner was found to correlate not only with BLV infection capacity as assessed by syncytium formation, but also with BLV-induced disease progression. The analyses described here show that the BLV-CoCoMo-qPCR method is a useful tool for evaluating BLV infection status. Conventional serological techniques, including AGID, PHA, and ELISA, are commonly used to diagnose BLV infection in Japan. Especially, the AGID test is currently a “golden standard” for determining BLV infection in Japan. We demonstrated that animals that were BLV-positive, as determined by the serological test (46.9% for PHA, 15.3% for AGID, and 62.3% for ELISA), were negative for proviral load, as determined by BLV-CoCoMo-qPCR (Table 2 and Figure 2). Furthermore, the sensitivity and reproducibility of BLV-CoCoMo-qPCR were greater than those of two previously developed real-time PCR methods (the TaqMan MGB assay developed by Lew *et al.* and the commercial TaKaRa cycleave PCR kit), using an infectious full-length molecular clone of BLV, pBLV-IF [24]. Moreover, the proportion of 370 cattle that were positive for anti-BLV antibody by the three serological tests was partially correlated with the proviral load determined by BLV-CoCoMo-qPCR. This result was confirmed by the finding that the kinetics of the proviral load did not quite correlate with changes in anti-BLV antibody production in two cattle experimentally infected with BLV. Collectively, these results suggest that the quantitative measurement of proviral load by BLV-CoCoMo-qPCR is a useful for monitoring the spread of BLV. In addition, the results show that serological and genomic tests complement each other and result in correct detection of BLV-infected cattle.

Whereas the positive detection ratefor nested PCR correlated well with the proviral load determined by BLV-CoCoMo-qPCR, the rates of animals that were positive for anti-BLV antibody by the three serological test did not correlate with the proviral load (Figure 2A). This finding indicates an inconsistency between the proviral load determined by BLV-CoCoMo-qPCR and the detection of BLV infection by serological tests. High positive rates for each serotest were observed in animals that were negative in BLV-CoCoMo-qPCR (Table 2 and Figure 2A). One possible explanation is that SK577, which was experimentally infected with BLV, produced antibodies against BLV but had a very low copy number of BLV throughout the experimental period (Figure 3). We previously reported that BLV-infected cattle retain a full-length proviral genome throughout the course of the disease [8]. Another in vivo dynamic study indicated that the turnover rate of infected cells is higher in BLV-infected sheep [31]. Based on the present data and previous results, we speculate that BLV-infected cells that express viral genes are eliminated by a strong anti-viral immune response; however, this would allow the cells that escape host immunity to survive, resulting in persistence of the virus in those cells throughout lifespan of the animal. Alternatively, it may be that BLV does not accumulate only in the peripheral blood used for BLV-CoCoMo-qPCR, but also in organs. We observed that numerous cattle that were negative for anti-BLV antibody by each serotest (72 animals for PHA, 26 animals for AGID, and 2 animals for ELISA) were positive for the provirus as determined by BLV-CoCoMo-qPCR (Figure 2). This result indicates that it is difficult to detect BLV infection by using the serological test alone. BLV infections without the detection of BLV antibodies by serological tests have been observed previously [32-36]. Thus, this result showed that, when viral gene expression is very low in BLV-infected cells, the infected cells can escape the immune response and survive, without evoking an immune response by viral protein production.

An advantage of the BLV-CoCoMo-qPCR method is that it uses degenerate primers designed from 52 individual BLV LTR sequences identified from 356 BLV sequences in GenBank. It also uses the CoCoMo algorithm that was developed specifically for the detection of multiple viral species [21]. It is possible that the degeneracy of the CoCoMo primers could be too high, which would reduce the concentration of primers specific for a particular target sequence and decrease the sensitivity of the assay. However, this issue did not arise in the present study: despite the use of degenerate primers, the sensitivity and reproducibility of BLV-CoCoMo-qPCR were greater than that of two previously developed real-time PCR methods (i.e., TaqMan MGB which was developed by Lew *et al.* and TaKaRa cycleave PCR) (Table 1 and Figure 1), as follow reasons. 1) To improve the sensitivity of our assay, we selected BLV-LTR (which is present at two copies per provirus) as the target of CoCoMo-qPCR. In contrast, TaqMan MGB developed by Lew *et al.* and TaKaRa cycleave PCR target the BLV *pol* and *tax* gene, respectively, which are present at only one copy per provirus. 2) The TaqMan probe was used to improve the sensitivity and specificity and to counter any drawbacks associated with high degeneracy. Importantly, the sequence of the BLV TaqMan probe, located between positions corresponding to two of the CoCoMo primers, was completely conserved among the 52 BLV variants. 3) A preliminary experiment demonstrated that the primer annealing regions of the TaqMan MGB assay developed by Lew *et al.* were variable in 10% of the 78 *pol* sequences selected from GenBank (on 2^nd^ October, 2010). This finding indicates that it is difficult to detect all of these variants. By contrast, use of degenerate primers allows for the detection of BLV sequence variants, including those that arise from mutations. Indeed, we previously demonstrated that this method can detect various BLV strains of broad geographical origins, including Japan, Peru, Bolivia, Chile, and the U.S.A. [21].

In the present study, we found that the propagation of and immunoresponses to BLV were different in two cattle that carried different *BoLA-DRB3* genotypes. This experiment may help direct future research into examining whether or not progression of BLV-induced diseases correlates with not only viral propagation, but also with host factors that are associated with the immune response. We previously reported that, in sheep experimentally infected with BLV, quantitative and/or qualitative aspects of the immunoresponse, production of neutralizing antibodies against BLV, and elimination of BLV depended on the particular allelic forms of the MHC class II molecules expressed by an individual and, in particular, on certain polymorphic amino acid residues in class II molecules [37,38]. Additional studies are required to define the mechanism of association between BLV-induced disease progression and MHC polymorphism.

Using BLV-CoCoMo-qPCR, we previously found an increase in the proviral load during disease progression [21]. This result strongly suggests that proviral load may be an excellent indicator for monitoring disease progression and for implementing segregation programs to minimize BLV transmission. One advantage of the proviral load measurement is that it can be used to follow the dynamics of BLV-infected cells in vivo. However, in the current study, proviral load was only determined in cell populations from peripheral blood. Because transformed phenotype of target lymphocytes for BLV is CD5^+^-B cells [39], it is easy to imagine that the proviral load in peripheral blood increases with disease progression. However, BLV can infect not only B cells, but also many other cell populations. It is still unknown which peripheral blood or organs maintain the BLV proliferation. In cattle harboring anti-BLV antibodies but lacking detectable BLV provirus in the blood, BLV may accumulate not only in the peripheral blood but also in organs. On the other hand, in cattle showing detectable BLV provirus in peripheral blood but lacking anti-BLV antibodies, BLV gene expression may be strongly suppressed. The BLV-CoCoMo-qPCR method can be used to investigate the mechanism by which BLV persists in vivo, by analyzing which organ is the key component in the maintenance of BLV proliferation.

## Conclusions

Recently, we developed the BLV-CoCoMo-qPCR system to detect various BLV strains with broad geographical origins. The BLV-CoCoMo-qPCR method is a useful tool for evaluating the progression of BLV-induced disease. In this study, BLV-CoCoMo-qPCR was found to be highly sensitive when compared with the real-time PCR–based TaqMan MGB assay developed by Lew *et al.* and the commercial TaKaRa cycleave PCR system. We observed that numerous cattle that were negative for anti-BLV antibody by each serotest were positive for the provirus as determined by BLV-CoCoMo-qPCR. By contrast, numerous animals that were BLV-positive by the serological test showed a negative proviral load by BLV-CoCoMo-qPCR. This result was confirmed by the finding that the kinetics of the proviral load did not quite correlate with changes in anti-BLV antibody production in two cattle experimentally infected with BLV. This result indicates that it is difficult to detect BLV infection by using the serological test alone. Collectively, these results suggest that the quantitative measurement of proviral load by BLV-CoCoMo-qPCR is a useful for monitoring the spread of BLV.

## Abbreviations

ATL: Adult T-cell leukemia; AGID: Agarose Gel Immuno-diffusion; BLV: Bovine leukemia virus; BoLA: Bovine leukocyte antigen; CoCoMo: Coordination of Common Motifs; EBL: Enzootic bovine leucosis; ELISA: Enzyme-linked immunosorbent assay; FAM: 5'-carboxyfluorescein; HTLV: Human T-leukemia virus; LTR: Long terminal repeat; MGB: Minor groove binder; PBMC: Peripheral blood mononuclear cell; PCR: Polymerase chain reaction; PHA: Passive Hemagglutination antigen; PL: Persistent lymphocytosis; qPCR: Quantitative real-time PCR.

## Competing interests

Non-financial competing interests.

## Authors’ contributions

MJ participated in real time-PCR and nested PCR, analyzed data and helped to draft of manuscript. ST participated in the experimental design, analyzed date and helped to draft the manuscript. HM experimented of real-time PCR. JK carried out experimentally infection with BLV of cattle. NK and TM experimented of AGID and ELISA. TO and TN experimented of AGID and ELISA. YA conceived the study, participated in experiments, participated in experimental design, coordinated experiments, and drafted the manuscript. All authors read and approved the final manuscript.

## Authors’ information

Mayuko Jimba (Ph.D.): Ph.D. student of The University of Tokyo and Junior Research Associate (JRA) in RIKEN. Present position is Postdoctoral Research of RIKEN. Shin-nosuke Takeshima (Ph.D.): ASI researcher of RIKEN and Associate professor of The University of Tokyo. Hironobe Murakami (Ph.D.,D.V.M): Visiting researcher of RIKEN and postdoctoral researcher of Japan Foundation for AIDS Prevention, Junko Kohara (Ph.D.,D.V.M): Hokkaido Animal Research Center. Naohiko Kobayashi (Ph.D.,D.V.M): Gifu prefectual livestoch research institute. Tamako Matsuhashi (Ph.D.): Gifu prefectual livestoch research institute. Takashi Ohmori (Ph.D.,D.V.M): Nippon institute for biological science. Tetsuo Nunoya (Ph.D.,D.V.M): Nippon institute for biological science. Yoko Aida (Ph.D.,D.V.M): Unit leader of RIKEN and Professor of The University of Tokyo.
